# Meningioma involving the superior sagittal sinus: long-term outcome after robotic radiosurgery in primary and recurrent situation

**DOI:** 10.3389/fonc.2023.1206059

**Published:** 2023-07-11

**Authors:** Michael Schmutzer, Benjamin Skrap, Jun Thorsteinsdottir, Christoph Fürweger, Alexander Muacevic, Christian Schichor

**Affiliations:** ^1^ Department of Neurosurgery, University Hospital, Ludwig-Maximilians-University (LMU), Munich, Germany; ^2^ European Radiosurgery Centre, Munich, Germany

**Keywords:** meningioma, superior sagittal sinus, robotic radiosurgery, microsurgery, sinus occlusion

## Abstract

**Objective:**

Treatment for meningiomas involving the superior sagittal sinus (SSS) is challenging and proved to be associated with higher risks compared to other brain locations. Therapeutical strategies may be either microsurgical (sub-)total resection or adjuvant radiation, or a combination of both. Thrombosis or SSS occlusion following resection or radiosurgery needs to be further elucidated to assess whether single or combined treatment is superior. We here present tumor control and side effect data of robotic radiosurgery (RRS) in combination with or without microsurgery.

**Methods:**

From our prospective database, we identified 137 patients with WHO grade I meningioma involving the SSS consecutively treated between 2005 and 2020. Treatment decisions were interdisciplinary. Patients underwent RRS as initial/solitary treatment (group 1), as adjuvant treatment after subtotal resection (group 2), or due to recurrent tumor growth after preceding microsurgery (group 3). Positive tumor response was assessed by MRI and defined as reduction of more than 50% of volume. Study endpoints were time to recurrence (TTR), time to RRS, risk factors for decreased survival, and side effects. Overall and specific recurrence rates for treatment groups were analyzed. Side effect data included therapy-related morbidity during follow-up (FU).

**Results:**

A total of 137 patients (median age, 58.3 years) with SSS meningiomas WHO grade I were analyzed: 51 patients (37.2%) in group 1, 15 patients (11.0%) in group 2, and 71 patients (51.8%) in group 3. Positive MR (morphological response) to therapy was achieved in 50 patients (36.4%), no response was observed in 25 patients (18.2%), and radiological tumor progression was detected in 8 patients (5.8%). Overall 5-year probability of tumor recurrence was 15.8% (median TTR, 41.6 months). Five-year probabilities of recurrence were 0%, 8.3.%, and 21.5% for groups 1–3 (*p* = 0.06). In multivariate analysis, tumor volume was significantly associated with extent of SSS occlusion (*p* = 0.026) and sex (*p* = 0.011). Tumor volume significantly correlated with TTR (*p* = 0.0046). Acute sinus venous thrombosis or venous congestion-associated bleedings did not occur in any of the groups.

**Conclusion:**

RRS for grade I meningiomas with SSS involvement represents a good option as first-line treatment, occasionally also in recurrent and adjuvant scenarios as part of a multimodal treatment strategy.

## Introduction

Meningiomas are tumors of the arachnoid cap cells and contribute to nearly 14%–19% of all primary intracranial tumors (1). The majority of meningiomas is located supratentorially at the convexity (35%), parasagittally (20%), the sphenoid ridge (20%), intraventricularly (5%), and the tuberculum sellae (5%), whereas about 15% are found infratentorially (2, 3). Meningiomas are divided into three histological grades according to the World Health Organization (WHO) 2016 classification (4).

Since the seminal work of Simpson (5), a complete surgical resection, including the dural attachments whenever possible, has been considered to be the best treatment in regard to local disease control.

However, complete resection of meningiomas involving the major venous sinuses, e.g., the superior sagittal sinus (SSS), is challenging. Discontinuity of the venous drainage of the brain (due to tumor growth, surgical maneuvers, or radiation) may lead to venous congestion, resulting in severe neurological deficits (6–9). The Sindou classification (**Table 1**) was introduced to categorize the relationship between the tumor and the SSS and possibly to guide the surgical strategy (6–9). In the attempt to lower morbidity, alternative treatment strategies such as radiosurgery either alone or combined with a subtotal microsurgical resection have been proposed (10–14).

**Table 1:** Sindou classification and sinus occlusion evaluation.

Sindou classification	
Grade	Description
I	Meningioma attached to the outer surface of the sinus wall
II	Lateral recess invasion
III	Lateral wall invasion
IV	Both entire lateral wall and sinus roof invasion
V	Sinus totally occluded; one wall free
VI	Sinus totally occluded
Sinus occlusion	
Grade	Occlusion rate (coronal slice)
Partial	0%–49%
Subtotal	50%–99%
Total	100%

Data comparing radiosurgical treatment outcomes in case of solely stereotactic radiosurgery or in combination with microsurgical resection in this particular location are scarce and often combine multiple histological grades (15).

Therefore, the aim of this study was to evaluate tumor control rates and neurological outcome in the so far largest homogeneous cohort of radiosurgically treated grade I meningiomas involving the SSS. We also aimed to evaluate the role of RRS as a primary therapy or as an adjunct after initial partial resection either in an adjuvant or salvage role.

## Methods

### Patient population and treatment parameters

From our prospective database, all patients treated for a meningioma between 2005 and 2020 involving the superior sagittal sinus were screened. For each patient, clinical charts, treatment data, follow-up notes, and neuroimaging studies were reviewed.

Therapy decisions were interdisciplinary in all cases. For patients undergoing primary radiosurgery, MR morphological aspects (16) that highly suggest a grade I meningioma, such as homogeneous contrast enhancement, smooth margins to surrounding brain tissue, and no hints of invasive growth patterns, needed to be fulfilled.

Indications for primary RRS were in line with the EANO guidelines for meningioma treatment: patient’s preference, radiological tumor growth/progression, and mild neurological symptoms (16). For the final analysis, histologically proven grade II and III meningiomas were excluded. Indications for RRS were also traced and patients were divided into three groups according to the treatment sequence:

#### Group 1

Primary radiosurgery without prior surgery

#### Group 2

##### Integrated treatment concept

Adjuvant radiosurgery after subtotal microsurgical resection (without signs of progression)

#### Group 3

Salvage radiosurgery due to radiological progression after subtotal microsurgical resection

The Cyberknife (Accuray Inc., Sunnyvale, CA, USA) used for RRS is a frameless, image-guided robotic system. The therapeutic radiation (photon) beam is generated by a 6-MV compact linear accelerator mounted on a six-axis robotic manipulator. In a typical RRS treatment, 100–200 non-isocentric, non-coplanar beams are used for radiosurgery. Intra-fraction patient motion is compensated by the automatic adaptation of beam directions based on stereoscopic X-ray images of the patient’s skull acquired periodically during treatment. Radiosurgical treatment data were reviewed, particularly the irradiated tumor volume and radiation doses. Radiation-induced complications such as edema or necrosis were also documented.

FU examinations [neurological as well as magnetic resonance imaging (MRI)] were performed after 6 months, every year for 2 years, and every 2 years thereafter.

### Magnetic resonance imaging protocol

All pre- and post-treatment neuroimaging studies were reviewed. In particular, the following parameters were analyzed: localization of the meningiomas (divided in anterior, middle, or posterior third of the SSS) (17), SSS invasion according to the Sindou classification (**Table 1**) (6–9, 18), and SSS occlusion rate (1) (**Table 1**) based on coronal contrast-enhanced T1 (CE-T1) and T2 sequences (divided into three groups). Tumor size was calculated and measured by volumetry. Manual segmentation of pre- and post-operative T2 and CE-T1 images was performed using the bumper tool of the Precision treatment planning software (Accuray Inc., Sunnyvale, CA, USA). Volume calculation of T2 and CE-T1 of meningiomas was performed by multiplying the sum of the tumor areas outlined on each transverse slice by the corresponding slice thickness.

### Response to therapy, time to recurrence

Tumor response was assessed by MRI and divided into four categories (**Table 2**): tumor shrinkage and/or no change in size were scored as locally controlled disease. To exclude temporary tumor swelling after radiosurgery (19), a local recurrence was only scored after two consecutive follow-ups when an increase in size was observed.

**Table 2:** Response to therapy.

Response to RRS treatment	
Minimal response	Volume reduction < 50%
Partial remission	Volume reduction > 50%
Complete remission	Volume reduction 100%
No response	No change in volume reduction

Time to recurrence (TTR) was calculated as the time between treatment and the second positive MRI for tumor growth.

### Neurological outcome analysis

Perioperative morbidity was determined according to all documented medical, neurological, and approach-related adverse events and differentiated as transient or permanent deficit.

All data were collected in accordance with the World Medical Association Declaration of Helsinki (20). All patients expressed their consent for the treatment. For this study, we obtained an approval of the institutional review board of the Ludwig-Maximilians-University in Munich (reference number 20-479).

### Statistical methods

The reference point of this study was the date of first therapy. Primary endpoints were TTR, functional outcome, and treatment toxicity. The significance of time to event data was assessed using the Cox proportional hazards model and the log-rank test. Results were tested by using a 2-way analysis of variance (ANOVA) and Student’s *t*-test. Particular recurrence rates for each treatment arm (groups 1–3) were defined as number of recurrences of each treatment arm/number of total patients per treatment arm. The overall recurrence rate was specified as the number of all recurrences/total patient number. For risk factor analyses, uni- and multivariate tests were conducted. Variables tested for predictive significance concerning local recurrence were age, sex, side of the tumor, tumor volume, and radiosurgical prescription dose. Further, ROC analyses were performed to examine the risk of recurrence depending on tumor volume. GraphPad PRISM8.0d (GraphPad Prism, San Diego, CA, USA) and Excel (Microsoft, Redmond, WA, USA) software were used for statistical analysis. Differences were considered statistically significant at *p* < 0.05.

## Results

### Patient characteristics and study population

**Figure 1:**
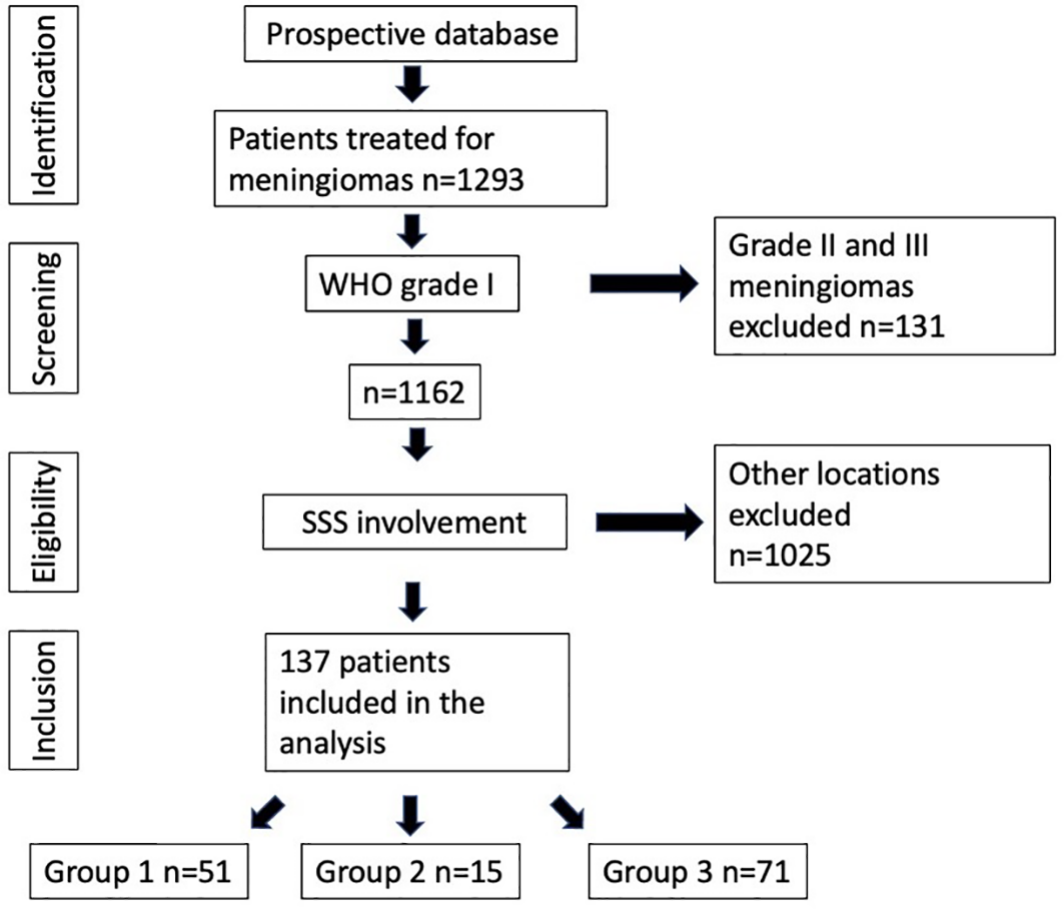
Patients with meningiomas treated at the European Radiosurgery Center.

**Table 3** summarizes patients’ characteristics in detail. In total, 1,293 patients with grade I meningiomas (*n* = 1162, 89.9%), grade II meningiomas (*n* = 123, 9.5%), and grade III anaplastic meningiomas (*n* = 8, 0.6%) were treated at our department. After exclusion of grade II and grade III tumors, 137 meningiomas (m:f = 1:2.3) involving the superior sagittal sinus were included in this study (**Figure 1**). Mean age at treatment was 57.4 years (median, 58.3 years; range, 25.7–87.1 years).

**Table 3:** Characteristics of patients.

**Number of patients**	137
**Number of tumors**	148
Localization (side)	
**Right (%)**	55 (40.1)
**Left (%)**	58 (42.3)
**Both sided (%)**	24 (17.5)
Localization at superior sagittal sinus	
**First ^1^/_3_**	18 (13.2)
**Mid ^1^/_3_**	82 (59.8)
**Last ^1^/_3_**	37 (27.0)
Sex	
**Male (%)**	42 (30.7)
**Female (%)**	95 (69.3)
**Median age (years)**	58.3 (25.7-87.1)
Treatment sequence	
**Group 1 (%)**	51 (37.2)
**Group 2 (%)**	15 (11.0)
**Group 3 (%)**	71 (51.8)
**Number of CK sessions**	152
Follow-up	
**Median (months)**	55.2 (3.4–200.3)
**≥1 year (%)**	112 (81.8)
**≥3 years (%)**	79 (57.7)
**≥5 years (%)**	46 (33.6)
**≥10 years (%)**	14 (10.2)
**Median time to recurrence (months)**	51.5 (3.4–200.3)
**Median tumor volume (cm^3^)**	5.8 (0.7–32.3)
**Median dose (Gy)**	15 (12–25)
**Median isodose (%)**	70 (60–75)

A total of 51 patients (37.2%) received a primary RRS (group 1), 15 patients (11.0%) were treated for a residual tumor after microsurgical resection (group 2), and 71 patients (51.8%) were treated due to relapse after surgery (group 3). Out of 86 patients of groups 2 and 3, 26 (30.2%) received surgery at the LMU University Hospital, whereas 60 patients (69.8%) underwent surgery at other institutions. Tumor volumes of patients operated at LMU University Hospital did not differ significantly from patients operated at external clinics (9.0 ± 5.4 cm^3^ vs. 7.1 ± 6.6 cm^3^, *p* = 0.17). In 18 (13.2%) patients, the tumor was located in the first third of the SSS, in 82 (59.8%) in the mid third, and in 37 (27.0%) in the last third of the SSS.

### Sinus occlusion and invasion

**Figure 2:**
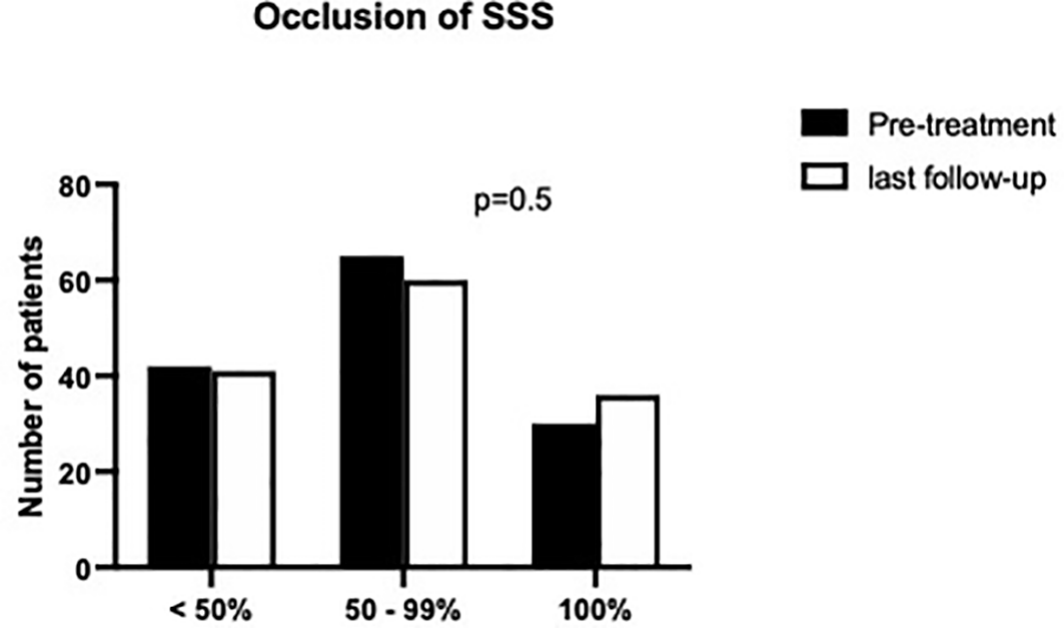
SSS occlusion rate prior to treatment and at the last follow-up.

**Figure 3:**
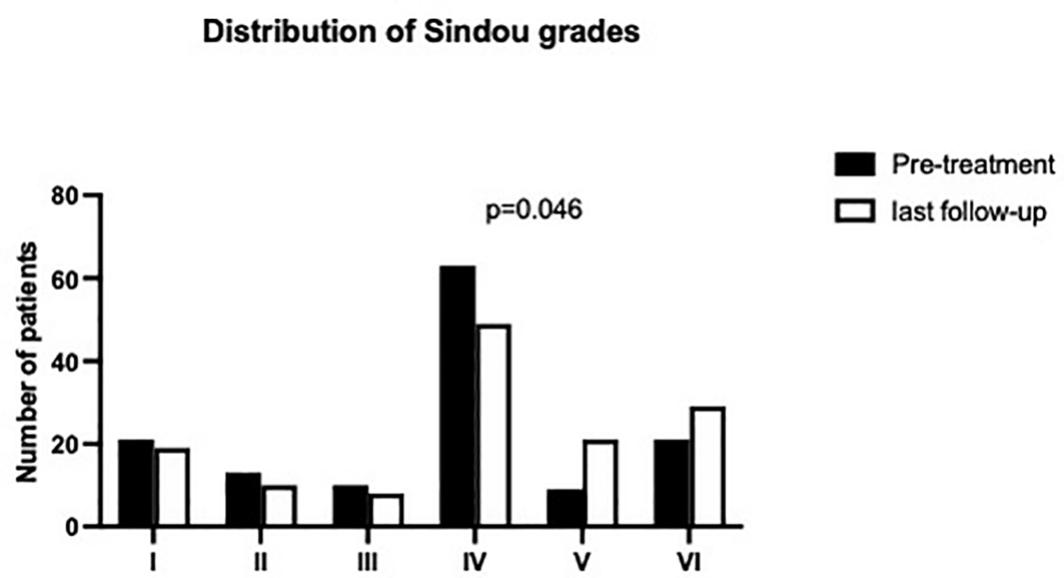
Distribution of Sindou grades prior to treatment and at the last follow-up.

The median SSS occlusion ratio was subtotal both before treatment and at last FU (see **Figure 2** for details). Sindou grade IV was median value for sinus invasion for both pre-treatment and at last FU (see **Figure 3**).

**Table 4** summarizes anatomical morphological classifications for meningiomas according to the defined treatment groups. For all treatment arms, we found a cumulative tumor localization in the mid third of the superior sagittal sinus (*p* = 0.051) and further a subtotal occlusion of the coronal diameter (*p* = 0.29), but without statistical significance. A predominant accumulation of Sindou grade IV (*p* = 0.046) throughout the treatment arms was noticed.

**Table 4:** Distribution of Sindou grades (I–VI), extent of SSS occlusion (<50%, 50%–99%, 100%), and SSS localization (first, middle, and last third) for patients of the three treatment groups.

Parameter	Group 1 *n* = 51	Group 2 *n* = 15	Group 3 *n* = 71	p-value
Sindou grade, *n* (%)				
**I**	13 (25.5)	2 (13.3)	6 (8.4)	0.78
**II**	4 (7.8)	1 (6.7)	8 (11.3)	0.8
**III**	3 (5.8)	1 (6.7)	6 (8.4)	0.77
**IV**	18 (35.3)	6 (40.0)	39 (54.9)	**0.046**
**V**	5 (9.8)	1 (6.7)	3 (4.2)	0.76
**VI**	8 (15.7)	4 (26.7)	9 (12.7)	0.78
SSS occlusion, *n* (%)				
**Partial**	18 (35.5)	4 (26.7)	20 (28.2)	0.76
**Subtotal**	20 (39.2)	6 (26.7)	39 (54.9)	0.29
**Total**	13 (25.5)	5 (33.3)	12 (16.9)	0.46
SSS localization, *n* (%)				
**First ^1^/_3_**	6 (11.8)	0	12 (16.9)	0.24
**Mid ^1^/_3_**	29 (56.9)	10 (66.7)	43 (60.6)	0.051
**Last ^1^/_3_**	16 (31.4)	5 (33.3)	16 (22.5)	0.26

Bold values are statistically significant.

### Treatment modalities and subgroups

Treatment of SSS meningiomas was either initial radiosurgery without prior surgical intervention (group 1), adjuvant RRS after subtotal resection without evidence of recurrence (group 2), or after local tumor recurrence of a surgical residue (group 3). All patients were treated with the CyberKnife® system (Accuray Inc., Sunnyvale, CA, USA). A total of 152 RRS sessions in 137 patients/tumors were performed. A total of 131 patients were treated with a single session and 6 patients underwent an average of 4.7 ± 0.8 therapy sessions (median, 5; range, 3–5). The mean target volume was 6.7 ± 5.3 cm^3^ (median, 5.81 cm^3^; range, 0.67–32.27 cm^3^) irradiated with an average dose of 15.5 ± 2.1 Gy (median, 15.0 Gy; range, 12.0–25.0 Gy). The applied dose and isodose on the three treatment groups did not differ significantly (*p* = 0.2 and 0.4).

**Tables 3**, **4** show detailed clinical parameters for the three treatment groups. **Table 5** highlights radiometrical data according to the three subgroups.

**Table 5:** Treatment and clinical parameters for patients in the three treatment groups.

Parameter	Group 1	Group 2	Group 3	p-value
**Age (years)**	54.7 ± 12.3	53.2 ± 12.7	60.2 ± 13.7	0.06
**Dose (Gy)**	15.9 ± 2.9	14.8 ± 1.3	15.4 ± 1.6	0.2
**Isodose (%)**	69.1 ± 2.2	69.0 ± 2.1	69.1 ± 2.6	0.9
**Time to recurrence (months)**	51.4 ± 40.1	51.6 ± 27.9	57.3 ± 32.3	0.2
**Volume (cm^3^)**	4.9 ± 4.2	10.8 ± 8.0	7.0 ± 4.7	**0.0004**

Bold values means that the p-value is less than 0.05 (p<0.05).

Patients irradiated with RRS due to a recurrent tumor after microsurgery (mean age at radiosurgery of 60.2 ± 13.7 years) tended to be older (*p* = 0.1) compared to patients undergoing RRS following microsurgery due to a residual tumor (53.2 ± 12.7 years) and to patients receiving initially RRS therapy (54.7 ± 12.3 years, *p* = 0.04). Tumor volume of primarily RRS-treated meningiomas was significantly lower with 4.9 ± 4.2 cm^3^ compared to recurrent and residual tumors (7.0 ± 4.7 cm^3^, *p* = 0.016 and 10.8 ± 8.0 cm^3^, *p* = 0.0004). Moreover, volumes of meningiomas in the recurrent or residual situation did differ significantly (10.8 ± 8.0 vs. 7.0 ± 4.7 cm^3^, respectively; *p* = 0.013). The average time to recurrence after RRS was lower for solely initial irradiated tumors (group 1) with 43.9 ± 41.9 months compared to radiosurgery-treated meningiomas due to recurrence (group 3) or a residual tumor (group 2) with 55.7 ± 47.9 months and 57.7 ± 33.9 months, respectively, but not statistically significant.

### Therapy-related morbidity and medical conditions during FU

Throughout the 157 RRS sessions, a transient peri-radiosurgical morbidity was observed in 8 patients (5.8%). These experienced perifocal post-radiation edema, which was symptomatic and required treatment with corticosteroids in 3 patients (2.2%) for a short period of time of less than 2 weeks. Two (1.4%) of these patients also developed radio-necrosis, but without the need of further interventions. These therapy-related morbidities did not differ significantly between groups 1 and 3. Detailed toxicity profile is listed in **Table 6**.

**Table 6:** Treatment-related toxicity and morbidities in the three subgroups.

Parameter	Group 1 *n* = 51	Group 2 *n* = 15	Group 3 *n* = 71	p-value
**Perifocal edema**	3 (2.2%)	1 (0.7%)	4 (2.9%)	0.9
**Radionecrosis**	0	1 (0.7%)	1 (0.7%)	0.2
**Headache**	4 (2.9%)	1 (0.7%)	5 (3.6%)	0.9
**Seizures**	1 (0.7%)	0	3 (2.2%)	0.6
**Vertigo**	0	0	3 (2.2%)	0.2

Ten (7.3%) patients reported headache during FU controls, three (2.2%) had seizures, which could be treated sufficiently with antiepileptic drugs, and two patients (1.4%) had vertigo and/or imbalance problems (see **Table 6**). Eight patients (5.8%) died during FU, but not due to meningioma- or RRS-related causes.

Interestingly, acute sinus venous thrombosis or venous congestion-associated bleedings did not occur in any of the groups, even if the sinus was already affected by the tumor, as classified by the Sindou- or the sinus occlusion criteria (**Table 4**).

### Follow-up and progression-free survival

The average FU period after RRS therapy was 55.2 ± 46.2 months (median, 42.0 months; range, 3.4–200.3 months). Average KPS at last FU was 97.3 (median, 100; range, 60–100).

A positive MRI morphological response to therapy was observed in 104 patients (75.9%). In 25 (18.2%) patients, no response was observed, and in 8 (5.8%) cases, there was evidence of progression (see **Table 7**).

**Table 7:** Radiological tumor response.

Response	*n* (%)
**Minimal response**	54 (39.4)
**Partial remission**	35 (25.5)
**Complete remission**	15 (10.9)
**No response**	25 (18.2)
**Progressive disease**	8 (5.8)

### Time to RRS therapy

The overall period between first diagnosis and RRS therapy initiation was 54.3 ± 49.8 months (median, 26.7 months; range, 0.4–305.3 months).

Patients with initial RRS-treated meningiomas (group 1) had an average period of 41.0 ± 35.4 months (median, 24.0 months; range, 1.1–194.5 months) between first diagnosis and RRS therapy initiation.

A total of 86 patients underwent 108 surgeries before RRS in the residual tumor (group 2) or local recurrent (group 3) (**Table 8**). For patients of group 2, the average time until RRS was 21.6 ± 15.8 months (median, 3.7 months; range, 0.4–63.9 months). In total, 15 patients received 18 microsurgical subtotal resections prior to radiosurgery.

**Table 8:** Overview of number of surgical procedures prior to RRS treatment.

Number of surgeries prior RRS	Group 2, *n* (%)	Group 3, *n* (%)
**1**	12 (11.1)	56 (51.8)
**2**	3 (2.8)	12 (11.1)
**3**	–	2 (1.8)
**4**	–	1 (0.9)

All patients of group 3 with a recurrent meningioma (*n* = 71) had 90 surgical procedures in total before RRS treatment. The average period between microsurgery and RRS for patients of group 3 was 67.4 ± 61.0 months (median, 40.9 months; range, 8.2–305.3 months).

### Time to recurrence

**Figure 4:**
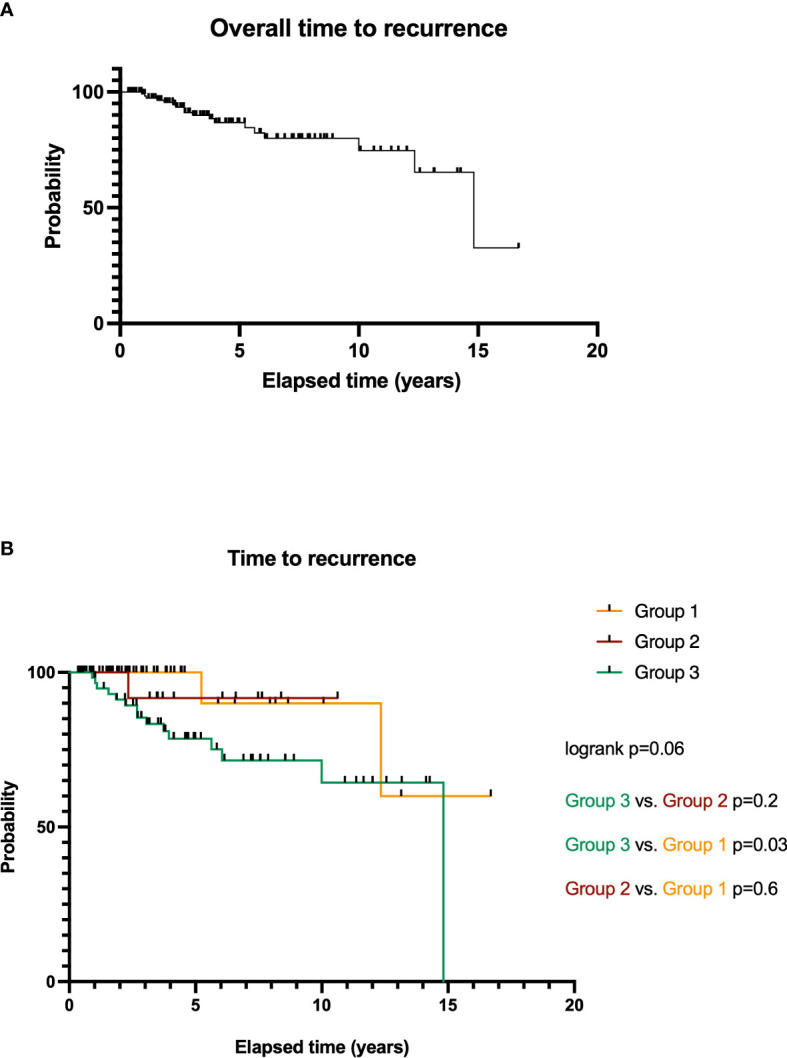
Overall time to recurrence (TTR) analysis for RRS-treated SSS meningiomas WHO grade I **(A)**. TTR of meningioma patients depending on treatment options: microsurgery and RRS after local tumor recurrence (group 3), microsurgery of a residual tumor (group 2), and initial RRS therapy (group 1, **(B)**).

Kaplan–Meier estimates are summarized in **Figure 4**. Eight patients died during FU. The average time to recurrence (TTR) was 51.5 ± 44.5 months (median, 41.6 months; range, 3.4–200.3 months). **Figure 4A** shows an overall TTR analysis regardless of treatment arms for all included patients: 1-, 2-, 5-, and 10-year probabilities without any tumor recurrences are 100%, 97.6%, 84.2%, and 59.4%. The recurrence rate for all patients undergoing RRS is 12.4% (17 patients). Overall, survival curves do not differ significantly from each other (log rank, *p* = 0.06).

Further particular analysis of TTR of the treatment groups is illustrated in **Figure 4B**.

Meningiomas treated in group 1 show an average TTR of 43.9 ± 41.9 months (median, 34.4 months; range, 5.1–200.3 months) with a treatment-related recurrence rate of 2.2% (3 patients). The 1-, 2-, 5-, and 10-year survival rates are 100%, 100%, 100%, and 90.0%.

For patients of group 2, average TTR is 57.7 ± 33.9 months (median, 44.4 months; range, 8.0–127.5 months). Here, the 1-, 2-, and 5-year survival rates were 100%, 100%, and 91.7%. Comparison of both surgery groups (groups 2 vs. 3, *p* = 0.2, HR = 3.3) and to group 1 is not significant (*p* = 0.6, HR = 1.8). The recurrence rate for this treatment arm after RRS is 1.5% (2 patients).

Patients of group 3 had an average TTR of 55.7 ± 47.9 months (median, 44.9 months; range, 3.4–177.8 months). The 1-, 2-, 5-, and 10-year probabilities without tumor recurrence were 98.3%, 91.2%, 78.5%, and 64.4%. The particular recurrence rate for meningiomas of group 3 is 8.8% (12 patients).

Here, survival curves do show a significant difference compared to the initial surgery cases: *p* = 0.03 (groups 3 vs. 1, HR = 4.3).

The recurrence rate for all patients undergoing RRS of meningioma involving the SSS is 12.4% (17 patients). Overall, survival curves do not differ significantly from each other (log rank, *p* = 0.06).

### Post-recurrence analysis after RRS

In total, 17 patients suffered from new recurrences of their treated meningioma. All recurrent tumors were again treated by RRS. The average post-recurrence survival for all recurrent tumors was 60.5 ± 35.2 months (median, 59.3 months; range, 5.6–133.6 months).

Of the 17 meningioma recurrences, 3 showed up in group 1, 2 in group 2, and 12 in group 3. Patients with recurrent tumors in groups 1–3 received further RRS sessions. The average post-recurrence survival is 34.1 ± 27.6 months (median, 35.9 months; range, 5.6–60.8 months) in group 1. In group 2, the average post-recurrence survival is 54.9 months, and in group 3, it is 68.0 ± 32.0 months (median, 68.3 months; range, 30.4–133.6 months).

### Risk analysis

In the uni- and multivariate risk analysis, various factors correlating significantly with tumor volume and TTR were found (see **Table 9**). The occlusion of the SSS (*p* = 0.026), male sex (*p* = 0.011), and the comparison of groups 1 vs. 3 (*p* = 0.0003) significantly correlated with tumor volume in the multivariate analysis. Further, tumor volume (*p* = 0.0046) and initial RRS therapy (group 1 vs. 2, *p* = 0.001) significantly correlated with TTR in the multivariate analysis.

**Table 9:** Uni- and multivariate analysis of factors influencing tumor volume and TTR.

Characteristic	Univariate 95% CI	p-value	Multivariate 95% CI	p-value
Tumor volume				
Occlusion of SSS	0.144 to 4.56	**0.0004**	0.39 to 6.25	**0.026**
Sindou classification	0.078 to 0.40	**0.0044**	−2.12 to 0.54	0.24
Localization SSS	0.045 to 2.93	0.15	−1.14 to 1.68	0.71
Sex (m vs. f)	0.13 to 0.45	**0.0005**	−4.57 to 0.60	**0.011**
Age	0.053 to 0.38	**0.011**	−0.002 to 0.01	0.06
Treatments				
Groups 1 vs. 2	−0.39 to 0.11	0.3	−0.03 to 0.23	0.2
Groups 2 vs. 3	−0.38 to 0.025	0.08	−0.45 to 0.37	0.4
Groups 1 vs. 3	−0.50 to 0.18	**0.0001**	−0.56 to 3.34	**0.0003**
TTR				
Occlusion of SSS	−0.22 to 0.13	0.56	−38.5 to 6.9	0.17
Sindou classification	−0.15 to 0.19	0.78	−3.0 to 16.7	0.17
Localization SSS	−0.25 to 0.09	0.38	−15.49 to 54.39	0.34
Sex (m vs. f)	−0.34 to 0.004	**0.045**	−18.07 to 11.35	0.65
Age	−0.22 to 0.13	0.58	−0.91 to 0.06	0.09
Tumor volume	0.08 to 0.41	**0.0045**	0.67 to 3.57	**0.0046**
Treatments				
Groups 1 vs. 2	0.80 to 1.1	**0.0001**	0.57 to 3.64	**0.001**
Groups 2 vs. 3	0.12 to 0.40	0.2	0.23 to 0.38	0.2
Groups 1 vs. 3	0.51 to 0.76	**0.0001**	0.47 to 0.76	0.5

Bold values means that the p-value is less than 0.05 (p<0.05).

The ROC analysis suggests for primary RRS-treated meningiomas (group 1) a maximum tumor volume of 5.1 cm^3^ as the cutoff value (*p* = 0.0004) for radiosurgical treatment to further reduce (in recurrent situations) tumor recurrence. Similarly, for meningiomas, which underwent initial surgical treatment, a tumor volume of more than 5.2 cm^3^ was associated with higher recurrence rates (*p* = 0.0004).

## Discussion

The management of parasagittal meningiomas, especially those involving the SSS, can still be challenging today. Historically, the optimal treatment was considered as an aggressive total Simpson grade 1 resection. This is achievable in only a minority of cases (21) and requires complex reconstructive techniques of the draining veins and SSS (7) with relevant morbidity and mortality (22). Even after radical resection, recurrence rates ranging from 4% (7) to 13% (23, 24) are reported. Kondziolka et al. (11) introduced the concept of multimodal treatment for parasagittal meningiomas. They advocated radiosurgery as a first-line treatment for meningiomas smaller than 3 cm and subtotal surgery (leaving the sinus part) followed by radiosurgery for bigger lesions. More recently, less aggressive surgical strategies have gained popularity (25–27), where precedence is given to a lower mortality and morbidity over total resection (leaving parts that are invading the sinus or growing in the sinus, especially if the sinus is not completely occluded). In a recent systematic review, Giordan et al. (22) compared the two surgical strategies. They showed that the non-aggressive surgical strategy achieved better functional outcomes and that, in the aggressive strategy group, the rate of postoperative venous infarcts was doubled. The overall recurrence rate was 7% for an FU of 4.9 years in the aggressive group and 13% for an FU of 6 years in the non-aggressive group. However, the benefits of an aggressive surgical strategy also remain unclear on the other side, because a recently published study by Wang et al. (28) reported a higher KPS score of patients who underwent an aggressive surgical tumor resection.

In a context of multimodality treatment, several studies have shown the role of stereotactic radiosurgery both as a first and as an adjuvant treatment following surgery (29–34).

### Study population

Many radiosurgical series present data regarding multiple locations and multiple histological (grading) diagnoses, and often many of the patients included could be considered “salvage” cases (35).

To our knowledge, this study presents the biggest consecutive series of WHO grade I (also presumptive) parasagittal meningiomas with SSS involvement treated at a single center in a 15-year interval. A total of 137 patients were analyzed. We allocated the patients into three treatment arms: primary RRS treatment (51 patients), RRS treatment after subtotal surgery (15 patients), and RRS treatment after post-surgical recurrence (71 patients).

### Primary treatment vs. adjuvant therapy

RRS as a primary treatment has gained more and more popularity especially for smaller lesions in a variety of locations and is nowadays part of the neurosurgical armamentarium (11, 29, 30, 33, 36, 37). In our study, 51 patients received RRS as a first-line treatment. Their TTR was 43.9 ± 41.9 months with 5- and 10-year survival rates of 100% and 90.0%, respectively, and a recurrence rate of 2.2%.

A total of 86 patients received RRS post-surgery, either when growth of the residual tumor occurred (71 patients, group 3) or after sub-total resection as adjuvant treatment (15 patients, group 2).

Group 2 had a time to recurrence of 57.7 ± 33.9 months, and the 1-, 2-, and 5-year probabilities without tumor recurrence were 100%, 100%, and 91.7%. Group 3 had a time to recurrence of 55.7 ± 47.9 months, and the 1-, 2-, 5-, and 10-year survival rates were 98.3%, 91.2%, 78.5%, and 64.4%. In our series, there was no statistically significant difference between the three arms.

The overall TTR regardless of treatment arm for all included patients was 100%, 97.6%, 84.2%, and 59.4% at respectively 1, 2, 5, and 10 years. Kondziolka et al. (11) reported a similar tumor control rate at 5 years of 93 ± 5% in the primary RRS group but a lower control rate of 60 ± 10% in the group of patients with previous surgical treatment. Also, DiBiase et al. (10) reported a 5-year disease-free survival of 86.2% for benign meningiomas at multiple locations and Park et al. (38) reported a 3-year recurrence free rate of 95% for subtotally resected meningiomas (all locations and grades) with additional RRS treatment. In the study of Colombo et al. (33) and Hadelsberg et al. (39), the tumor control rate from benign primarily RRS-treated meningiomas (multiple locations) was 93.56% and 90.6%, respectively.

### Timing of RRS

The role of adjuvant radiotherapy for grade II and III meningiomas is supported by a number of studies (40). There is also some evidence supporting upfront adjuvant radiotherapy (41). Its role for grade I meningiomas is still discussed. Some authors report no benefit of the adjuvant therapy, and therefore, the timing of the treatment remains unclear (42). In our series, a positive MRI morphological response to therapy was observed in 50 patients (36.4%). Two strategies are usually proposed: an early treatment of the residue or an initial wait-and-see approach with serial MRI follow-ups with secondary treatment (in this case RRS) reserved only when recurrence is observed. Some studies support the role of an early postoperative radiosurgical treatment in improving PFS compared to a treatment at recurrence (11, 43–45). Frostell et al. (46) published a study in which they confronted adjuvant radiosurgical treatment after subtotal resection of meningiomas involving the SSS. Their results showed better outcome for patients treated with upfront radiosurgery compared to those treated for recurrence. In their study, however, meningiomas of all three grades were included.

Also, our group (47) recently presented a study where early postoperative radiation treatment was advantageous in spheno-orbital grade I meningiomas. In our study, we found no statistically significant difference in PFS between the two postsurgical groups (post-recurrence and post-subtotal). It is important to notice that the non-significance of these data could be due to the relatively small number of patients that underwent RRS directly after subtotal resection (15 patients). However, Pikis et al. (48) showed that upfront stereotactic radiosurgery of grade I parasagittal meningioma leads to superior radiological tumor control compared to watch and wait. The question of the ideal timing for a post-surgical RRS therapy in a multimodal treatment remains open and needs to be further elucidated.

### Tumor volume vs. recurrence

Tumor volume plays an important role in both rate of recurrence and complication rate post-radiosurgery (10, 49). This was also confirmed in our study.

ROC analysis regarding tumor volume and risk of recurrence showed that a volume greater than 5.1 cm^3^ and 5.2 cm^3^ in the primarily treated and postsurgical RRS groups did correlate to higher rate of recurrence. DiBiase et al. (10) and Kondziolka et al. (11) described bigger tumor volumes, >10 cm^3^ and >7.5 cm^3^, respectively, correlating with local recurrence.

In our opinion, the volume analysis represents a valuable tool in guiding the choice both of the primary treatment as of the timing of post-surgical RRS therapy. Furthermore, it should be used in the planning of the surgical strategy in a multimodal treatment environment.

Radiosurgical treatment can be associated with treatment-related morbidity. Former studies reported of patients with parasagittal meningiomas having higher risks of developing post-radiation edema compared to skull base meningiomas (50, 51). In our series, eight patients (5.8%) experienced perifocal post-radiation edema. Only three patients were symptomatic, requiring low-dose dexamethasone treatment, and two of these patients also developed radio-necrosis in follow-up MRI. This is less than what other studies reported (52). No patient needed a surgical treatment for a radiosurgical complication and no patient experienced an SSS thrombosis following RRS.

## Limitations

The main limitation of this study is its design as a retrospective cohort study. Furthermore, the three treatment arms are imbalanced due to different numbers of patients. Moreover, the allocation of patients to one of the arms was not randomized but based on clinical decision.

For the primary RRS-treated patients, the diagnosis of grade I meningioma was based solely on radiological criteria and not on a histological confirmation.

## Conclusion

RRS for parasagittal grade I meningiomas with SSS involvement represents a good option as a first-line treatment, but also a second-line treatment in a recurrent and adjuvant (post-subtotal resection) scenario. Furthermore, it plays an important role in a multimodal strategy for the treatment of meningiomas involving the SSS.

## Data availability statement

The original contributions presented in the study are included in the article/supplementary material. Further inquiries can be directed to the corresponding author.

## Ethics statement

The studies involving human participants were reviewed and approved by Institutional review board of the Ludwig-Maximilians-University in Munich (reference number 20-479). Written informed consent for participation was not required for this study in accordance with the national legislation and the institutional requirements.

## Author contributions

Conception and design: MS, AM, and CS. Acquisition of data: MS and BS. Analysis and interpretation of data: MS, BS, JT, and CS. Drafting the article: MS. Critically revising the article: all authors. Reviewed submitted version of manuscript: all authors. Approved the final version of the manuscript on behalf of all authors: MS. Statistical analysis: MS. Study supervision: AM and CS.
